# Even–odd layer-dependent magnetotransport of high-mobility Q-valley electrons in transition metal disulfides

**DOI:** 10.1038/ncomms12955

**Published:** 2016-09-21

**Authors:** Zefei Wu, Shuigang Xu, Huanhuan Lu, Armin Khamoshi, Gui-Bin Liu, Tianyi Han, Yingying Wu, Jiangxiazi Lin, Gen Long, Yuheng He, Yuan Cai, Yugui Yao, Fan Zhang, Ning Wang

**Affiliations:** 1 Department of Physics and the Center for 1D/2D Quantum Materials, the Hong Kong University of Science and Technology, Hong Kong, China; 2 Department of Physics, University of Texas at Dallas, Richardson, Texas 75080, USA; 3 Beijing Key Laboratory of Nanophotonics and Ultrafine Optoelectronic Systems, School of Physics, Beijing Institute of Technology, Beijing 100081, China

## Abstract

In few-layer transition metal dichalcogenides (TMDCs), the conduction bands along the ΓK directions shift downward energetically in the presence of interlayer interactions, forming six Q valleys related by threefold rotational symmetry and time reversal symmetry. In even layers, the extra inversion symmetry requires all states to be Kramers degenerate; whereas in odd layers, the intrinsic inversion asymmetry dictates the Q valleys to be spin-valley coupled. Here we report the transport characterization of prominent Shubnikov-de Hass (SdH) oscillations and the observation of the onset of quantum Hall plateaus for the Q-valley electrons in few-layer TMDCs. Universally in the SdH oscillations, we observe a valley Zeeman effect in all odd-layer TMDC devices and a spin Zeeman effect in all even-layer TMDC devices, which provide a crucial information for understanding the unique properties of multi-valley band structures of few-layer TMDCs.

## Abstract (web summary)

Few-layer transition metal dichalcogenides exhibit strong spin-valley entanglement and unconventional quantum Hall states, however their study has been limited by electron mobility. Here, the authors explore how quantum transport varies between even- and odd-layered systems of high mobility.

## Graphical abstract

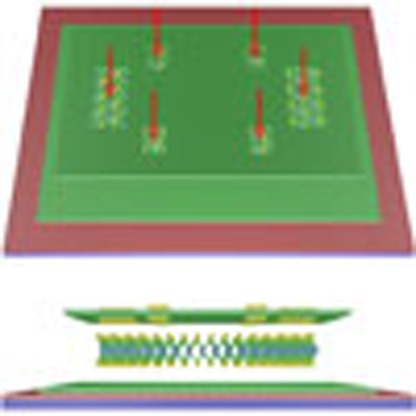

Strong spin–orbit couplings in monolayer transition metal dichalcogenides (TMDC)^1^,^2^,^3^,^4^ entangle the spin and valley degrees of freedom of the valence band states at K valleys^5^,^6^. This gives rise to exciting electronic and excitonic properties, such as optical circular dichroism^7^,^8^,^9^,^10^, opto-valley Hall effect^11^,^12^ and optical valley Zeeman effect^13^,^14^,^15^,^16^,^17^,^18^. Inversion symmetry breaking in monolayer TMDCs plays an important role in opening energy gaps at the energy range of visible lights at K valleys^7^,^8^,^9^,^10^. Together with the substantial spin–orbital couplings, the broken inversion symmetry in monolayer TMDCs further splits the spin-up and spin-down bands at the valence band edge of K valleys, which is absent in any bilayer TMDC^10^,^19^. The former feature leads to the optical circular dichroism and valley Hall effect, the latter feature yields the observation of optical valley Zeeman effect. So far, the observed moderate electron mobility in most atomically thin TMDCs has prohibited the exploration of the quantum transport properties. Impurity scattering and non-ohmic contacts are two major obstacles to fabricating high-mobility TMDC-based field-effect (FE) devices. Several attempts have been made recently in improving the electrical contacts by, for example, using phase engineering^20^, graphene on MoS_2_ (ref. 21), Pt electrodes^22^ or selective etching process^23^.

Here we present a magnetotransport study of both even- and odd-layer TMDCs with hexagonal boron nitride (BN) encapsulated structures and metal contacts made by the selective etching process. As we will show, the peculiar electronic properties can be extended from monolayer TMDCs to few-layer TMDCs, from K valleys to Q valleys^24^, and from optical probe to transport detection. This possibility can be explicitly appreciated by comparing the quantum transport measurements in odd-layer and even-layer TMDCs, in which the essential inversion symmetry is broken and respected, respectively. The unique transport properties observed in BN-encapsulated TMDC devices provide useful information for a better understanding of the thickness dependent magnetotransport of high-mobility Q-valley electrons and the spin-valley entangled unconventional quantum Hall (QH) effects in atomically thin TMDCs.

## Results

### High-mobility TMDC devices

**Figure 1:**
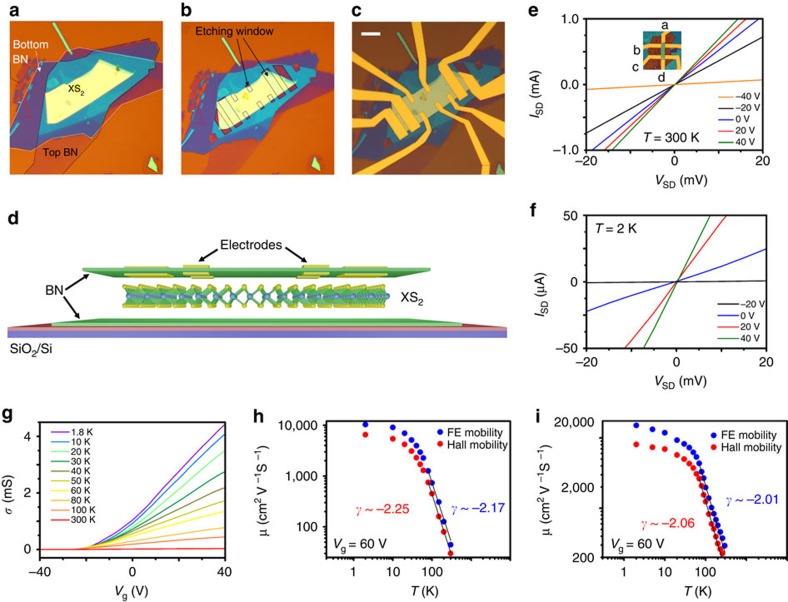
BN-TMDC-BN heterostructure device. (**a**) The sandwiched TMDC heterostructure. (**b**) The BN-TMDC-BN heterostructure for selective etching. The etching window is marked by arrows. (**c**,**d**) Optical (**c**) and schematic image (**d**) of a BN-TMDC-BN FE transistor device with a Hall bar configuration. Scale bar, 10 μm. (**e**,**f**) Two-terminal *I*_SD_−*V*_SD_ characteristics of a representative MoS_2_ device at 300 K (**e**) and 2 K (**f**). Linear *I*–*V* behaviour is observed in both cases. (**g**) Four-terminal conductance in the WS_2_ device plotted as a function of the gate voltage at various temperatures. (**h**,**i**) FE mobilities and Hall mobilities of MoS_2_ (**h**) and WS_2_ (**i**) at *V*_g_=60 V at various temperatures.

As discussed in our previous work^23^, to eliminate any impurity effects induced during device fabrication, we employ a polymer-free dry transfer technique^25^,^26^ in an inert environment of argon or nitrogen, as schematically demonstrated in Fig. 1a–d. Using the encapsulation of few-layer TMDCs in BN sheets and the selective etching process, we can achieve high-quality low-temperature ohmic contacts (0.15–0.5 kΩ·μm) and ultrahigh FE mobilities (10,500–19,600 cm^2^ V^−1^ s^−1^) in TMDC channels. For example, Fig. 1e,f show the *I*_SD_–*V*_SD_ curve of a nine-layer (9L) MoS_2_ device, where *V*_SD_ is the voltage source and *I*_SD_ is the measured current. The linear characteristic of this device is observed at both 300 and 2 K. The contact resistivity at *T=*2 K is ∼0.25 kΩ·μm (see details in Supplementary Fig. 1a–c for another 6L WS_2_ device).

The high quality of our BN-TMDC-BN heterostructures is reflected by their four-terminal FE mobilities 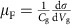, measured at different temperatures, where *σ* is the conductivity and *C*_g_ is the gate capacitance (1.1–1.2 F cm^−2^, as calculated based on the thickness of SiO_2_ (300 nm) and the bottom BN layer (15–50 nm)). The FE characteristics of 9L MoS_2_ (Fig. 1g) and 6L WS_2_ (Supplementary Fig. 1d) are shown, whose FE mobilities at room temperature are ∼50 cm^2^ V^−1^s^−1^ and ∼300 cm^2^ V^−1^ s^−1^, respectively. At *T*=2 K, our TMDC devices show excellent performance with remarkably improved FE mobilities (*μ*_F_∼10,500 cm^2^ V^−1^ s^−1^ for 9L MoS_2_ (Fig. 1h) and *μ*_F_∼16,000 cm^2^ V^−1^ s^−1^ for 6L WS_2_ (Fig. 1i)). The phonon scattering is suppressed, and the corresponding Hall mobilities (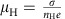) reach 6,700 and 8,000 cm^2^ V^−1^ s^−1^, where *n*_H_ is the carrier density obtained from the Hall measurement and *σ* is the conductivity. The FE mobility in our 6L WS_2_ device (∼16,000 cm^2^ V^−1^ s^−1^) is more than 30 times higher than the previously reported record for WS_2_ (∼486 cm^2^ V^−1^ s^−1^) (ref. 27). It must be noted that the screening effect may affect the back gating effect and produce inhomogeneous charge density across the layers^28^. This can give an inaccurate *C*_g_ and hence explain the difference between our Hall and FE mobilities.

### Quantum oscillations in odd-layer TMDCs

**Figure 2:**
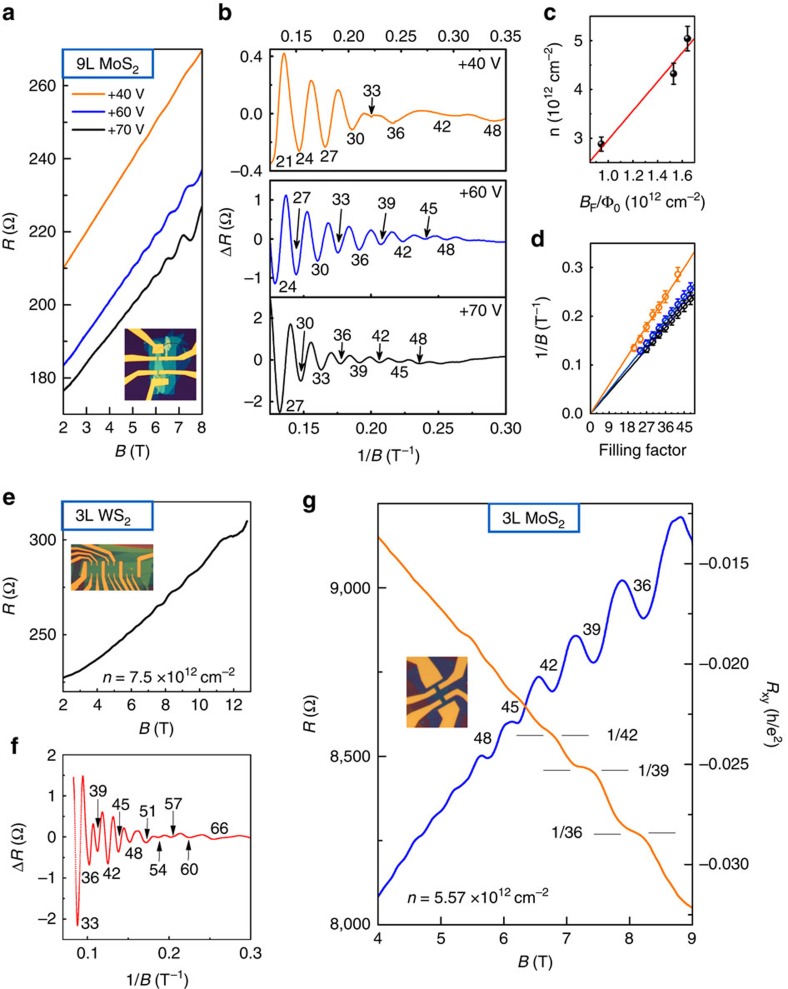
Quantum oscillations in odd-layer TMDCs. (**a***–***d**) Quantum oscillations in 9L MoS_2_. (**a**) Resistance *R* as a function of *B* field at + 40 V (orange line), +60 V (blue line) and +70 V (black line) gate voltages. The inset shows the sample image. (**b**) After subtracting the baselines of *R* ∼ *B* curves in **a**, Δ*R* curves plotted as a function of 1/*B* yields an oscillation period 1/*B*_F_, which decreases with increasing gate voltages. The filling factors are labelled for the oscillations valleys. The degeneracy of 6 arises from the degeneracy between the 3 Q and 3 Q' valleys; the spin degeneracy within each Q or Q' valley is already lifted by the broken inversion symmetry. At relatively high magnetic fields, an LL sextet can be lifted into two LL triplets caused by the valley Zeeman effect. (**c**) The total carrier density n obtained from the Hall measurements as a function of *B*_F_/*Φ*_0_ (black dots) for different gate voltages. The best fit (red line) indicates a LL degeneracy of ∼3.0±0.1. (**d**) LL filling factors as a function of 1/*B* at different gate voltages. The linear fit yields a zero berry phase. (**e**,**f**) Quantum oscillations in 3L WS_2_. (**e**) *R* plotted as a function of *B* at the carrier density of 7.5 × 10^12^ cm^−2^ (**f**) Δ*R* curves plotted as a function of 1/*B*. The LL degeneracy evolves from 6 at low-*B* fields to 3 at high-*B* fields. (**g**) The onset of QH states in 3L MoS_2_. Magnetoresistance resistance *R* (blue line) and Hall resistance *R*_*xy*_ (orange line) as a function of *B* field at 2 K. The QH states are shown by at least three almost quantized plateaus in *R*_*xy*_ at *ν*=36, 39 and 42.

In the representative 9L MoS_2_ device, the Shubnikov-de Hass (SdH) oscillations in the longitudinal resistance *R* appear at perpendicular magnetic fields *B*>4 T (Fig. 2a). This property is the hallmark of the high quality and homogeneity of our BN-MoS_2_-BN devices. Pronounced SdH oscillations are observed at relatively high gate voltages, where *μ*_H_ is sufficiently high. Quantitatively, at the low magnetic field range, the SdH oscillations in the longitudinal resistance *R* of a single sub-band in two-dimensional electron gas can be described by the Lifshitz–Kosevich formula^29^:


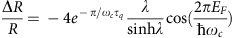


where *λ*=2π^2^*k*_B_*T*/ℏ*ω*_c_. The cyclotron frequency is given by *ω*_*c*_=e*B*/m*. *τ*_*q*_ is the quantum scattering time and the Fermi energy is described by *E*_F_=2πℏ^2^*n*/g_v_g_s_m*. *k*_B_ is the Boltzmann's constant, *T* the temperature, ℏ the plank constant, *e* the electron charge, *B* the magnetic field, m* the cyclotron mass of carriers, *n* the charge carrier density, *g*_v_ the valley degeneracy and *g*_s_ the spin degeneracy. In a two-dimensional electron gas, the SdH oscillations can display useful information about the quantization of Landau levels (LLs) when plotted versus 1/*B*. Figure 2b shows the plots of Δ*R* (that is, the background has been subtracted from *R*) as a function of 1/*B* at different gate voltages *V*_g_. The equal spacing between SdH valley positions implies the single-band nature at the studied *V*_g_. Thus, extracting further information using the Lifshitz–Kosevich formula is appropriate. In principle, the periodicity of SdH oscillations is 1/*B*_F_=*g*/Φ_0_*n*, where *g*=*g*_s_ × *g*_v_ is the LL degeneracy and Φ_0_=*h*/*e* is the flux quantum. At relatively high fields, the best fit of *n* versus *B*_F_/Φ_0_ (Fig. 2c) yields *g*=3.0±0.1; the linear fit of the LL filling factors versus the SdH valley positions (Fig. 2d) yields a zero Berry phase (the fitting results are in the range of 0.03±0.05 *π*). As Fig. 2b shows, the filling factors *ν*=36, 42 and 48 are also clearly observed at relatively low fields with a gate voltage *V*_*g*_=40 V. The degeneracy of 6 arises from the degeneracy between the 3 Q and 3 Q' valleys because the spin degeneracy within each Q or Q' valley is already lifted by the broken inversion symmetry in an odd-layer TMDC. At relatively high magnetic fields, an LL sextet can be lifted into two LL triplets caused by the valley Zeeman effect, which is similar to the K/K' valley Zeeman effects observed most recently using optical circular dichroism^13^,^14^,^15^,^16^. The Lande factor *g*_L_ can be roughly estimated using the formula *g*_L_*μ*_B_*B*_c_=*k*_B_*T*_C_, where *μ*_B_ is the Bohr magneton, and *B*_C_ is the lowest field and *T*_C_ is the highest temperature for our observation of the valley Zeeman effect^30^,^31^. With a filling factor of 33 at *V*_g_=40 V, the valley Zeeman splitting disappears at ∼10 K (see Supplementary Fig. 2a) amounting to *g*_L_∼3.4, which is comparable to those reported for WSe_2_ and MoSe_2_ monolayers. At relatively high gate voltages (60 and 70 V), where the Hall mobility is sufficiently high, the Zeeman effect takes place at a small strength of *B* field. The LL triplets start to appear where the SdH oscillations emerge.

The cyclotron mass of charge carriers in the 9L MoS_2_ device is obtained by investigating the temperature dependence of Δ*R* oscillations (see Supplementary Fig. 2a). For a given *E*_F_ (*V*_g_=60 V or *n*=4.32 × 10^12^ cm^−2^) and a given *B*, the Δ*R* peak amplitudes (see Supplementary Fig. 2b) follow the Ando formula^32^ 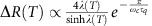. We obtain m*≈0.27±.01*m*_e_, which is smaller than the effective mass (∼0.5*m*_e_) obtained by our density functional theory (DFT) calculations. Depending on the thickness of odd-layer samples, the measured m* can vary from 0.3 to 0.4*m*_e_ partially due to the uncertainty of the measured temperature. The corresponding quantum scattering time is *τ*_q_=206 fs, which is much shorter than the transport scattering time *τ*_t_=(m*)/(*R*_0_e^2^*n*)=1,100 fs, thereby demonstrating that long-range scattering is dominant in our MoS_2_ sample (see Supplementary Fig. 3).

As expected, the valley Zeeman effect can also be observed in a 3L WS_2_ sample (Fig. 2e,f). After subtracting the background of the data shown in Fig. 2e, we plot Δ*R* as a function of *B* (Fig. 2f). At high-*B* fields, the LLs developed from sextets to triplets, with increasing amplitudes of SdH oscillations. Remarkably, the ultrahigh mobility achieved in these TMDC samples even enables us to observe the onset of the QH effect. Figure 2g shows the longitudinal resistance *R* and Hall resistance *R*_*xy*_ as a function of *B* at 2 K in a 3L MoS_2_ device. Beyond 6 T, *R*_*xy*_ exhibits at least three well-quantized plateaus (*ν*=36, 39 and 42), and they match very well with the corresponding *R* valleys. As in other TMDC devices with an odd number of layers, the SdH oscillations in the 3L MoS_2_ device clearly exhibit an LL degeneracy of 3, implying the valley Zeeman splitting.

### Quantum oscillations in even-layer TMDCs

**Figure 3:**
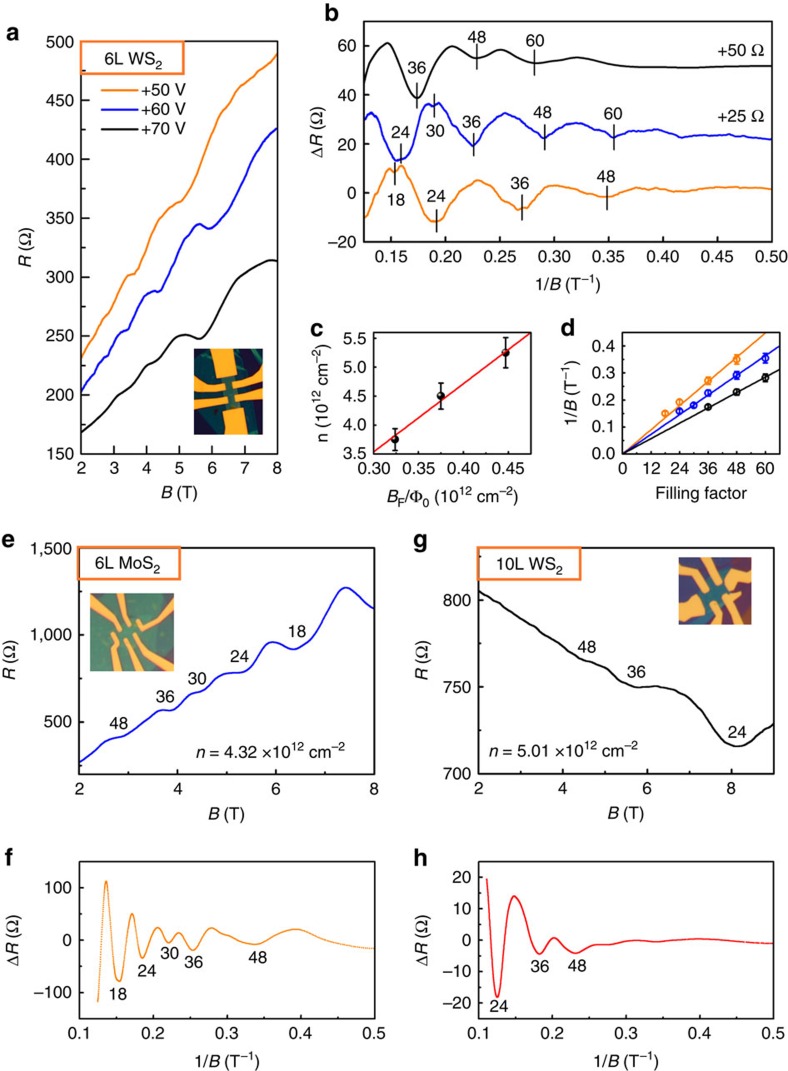
Quantum oscillations in even-layer TMDC. (**a***–***d**) Quantum oscillations in 6L WS_2_. (**a**) Resistance *R* as a function of *B* field at + 50 V (orange line), +60 V (blue line) and +70 V (black line) gate voltages. The inset shows the sample image. (**b**) Δ*R* plotted as a function of 1/*B* field yields an oscillation period 1/*B*_F_. The filling factors are labelled for the oscillation valleys. A twelve-fold LL degeneracy at low fields and six-fold LL degeneracy at high fields is observed, caused by the spin Zeeman splitting within each valley. (**c**) The total carrier density *n* obtained from the Hall measurements as a function of *B*_F_/Φ_0_ (black dots) for different gate voltages. The best fit (red line) indicates a LL degeneracy of ∼11.8±0.1. (**d**) LL filling factors as a function of 1/*B* for different gate voltages. The linear fit yields a zero Berry phase (the fitting results are in the range of −0.1 to+0.3 π) (**e**,**f**) Quantum oscillations in 6L MoS_2_. (**e**) *R* plotted as a function of *B* at the carrier density of 4.32 × 10^12^ cm^−2^ (**f**) Δ*R* curves plotted as a function of 1/*B*. The LL degeneracies derive from 12 at low-*B* fields to 6 at high-*B* fields. (**g**,**h**) Quantum oscillations of 10L WS_2_ show a LL degeneracy of 12. The negative magnetoresistance implies the existence of disorders, which might be the reason for the absence of the sixfold LLs at high-*B* fields.

The SdH oscillations in the representative 6L WS_2_ device emerge when *B* field is greater than 2.5 T (Fig. 3a). Although the gate voltages applied (*V*_g_=50–70 V) to the 6L WS_2_ device are similar to those applied (*V*_g_=40, 60 and 70 V) to the 9L MoS_2_ device, the period of SdH oscillations appears twice larger in the 6L WS_2_ device (Fig. 3b). Given that the experimentally accessible carrier density is low, the Fermi energy crosses only the lowest spin-degenerate sub-band at the Q/Q' valleys in our calculated band structure of 6L WS_2_. The single sub-band nature is also evidenced by the unique period in the SdH oscillations (Fig. 3b). The linear fit of *n* versus *B*_F_/Φ_0_ (Fig. 3c) indicates a LL degeneracy of ∼11.8±0.1; the linear fit of the LL filling factors versus the SdH valley positions (Fig. 3d) yields a zero Berry phase. At a large field of 6.5 T, the secondary SdH valleys and doubling of the oscillation frequency are clearly visible because of the spin Zeeman splitting of LL duodectets into LL sextets (see Supplementary Fig. 4). The disappearance of secondary SdH valleys at around 10 K further indicates that the Lande factor is *g*_L_∼2.2 (Supplementary Table 1). Under similar experimental conditions, the presence of SdH valleys, as a result of the complete filling of a LL duodectet or sextet, has been repeatedly observed; for example, in a 6L MoS_2_ device (*g*=12/6 at low-/high-*B* fields in Fig. 3e,f), in a 10L WS_2_ device (*g*=12 in Fig. 3g,h) and in a 10L MoS_2_ (*g*=6 in Supplementary Fig. 5). Clearly, in contrast to odd-layer MoS_2_ devices (for example, 3L and 9L MoS_2_), even-layer MoS_2_ devices exhibit doubled LL degeneracies (see for example the data of 6L MoS_2_ in Fig. 3). We note that a tilted magnetic field will be helpful to further exploration of the TMDC QH effect and better determination of the physical parameters.

The cyclotron mass m* in 6L WS_2_ is also investigated (See Supplementary Fig. 4). On the basis of the Δ*R* data plotted as a function of *B* and *k*_B_*T* at *V*_g_=70 V (*n*=3.75 × 10^12^ cm^−2^), we obtain m*≈0.20±0.04*m*_e_. This indirect experimental value of m* is again smaller than the effective mass ∼0.5*m*_e_ obtained in our DFT calculations. We noted that the limited amplitude of SdH oscillations and sample temperature uncertainty may cause certain deviation of m*. On the basis of the Ando formula, we further obtain the quantum scattering time *τ*_q_=586 fs in the 6L WS_2_ device, which is smaller than the corresponding transport scattering time *τ*_t_=1,300 fs (see Supplementary Fig. 6).

### Spin-valley coupled Q valleys in few-layer TMDCs

**Figure 4:**
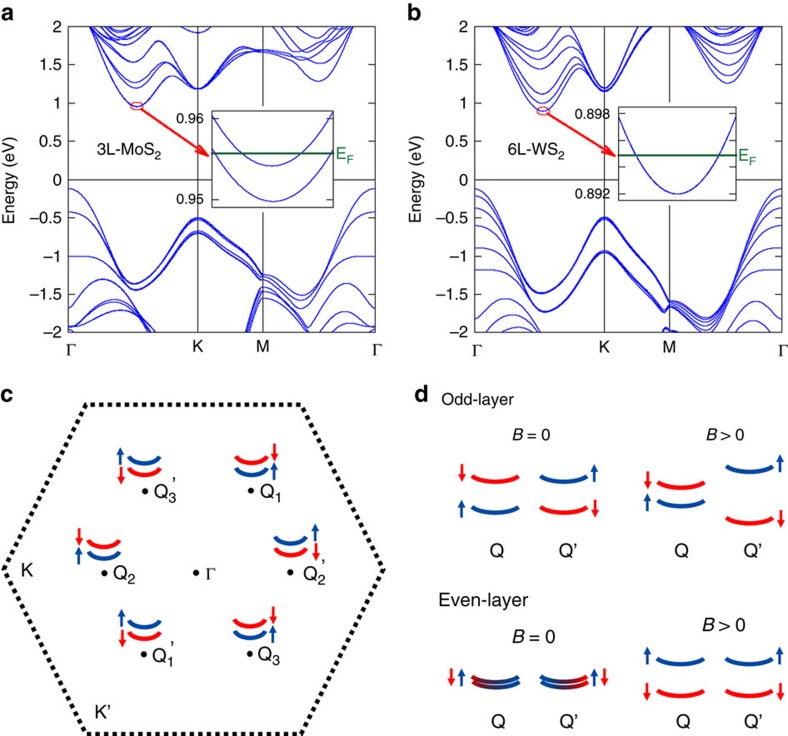
Layer-dependent spin-valley coupled Q valleys in TMDCs. (**a**) Calculated band structure of 3L MoS_2_. The bottom of conduction band is located at the Q (Q') valleys. At the edge of each Q (Q') valley, the Fermi level only crosses the lowest non-degenerate sub-band, whose spin-up and spin-down sub-bands are lifted by 4.3 meV. (**b**) Calculated band structure of 6L WS_2_. The energy bands are spin-degenerate at the edge of each Q (Q') valley. These spin-valley coupled band edges are further illustrated in (**c**), where the red and blue colours denote the spin-down and spin-up bands, respectively. Q_1_, Q_2_ and Q_3_ have the same spin, and Q_1_', Q_2_' and Q_3_' are their time reversals. (**d**) Schematic diagrams for the Bloch bands, showing the valley Zeeman effect in odd-layer devices and the spin Zeeman effect in even-layer devices. For odd-layer samples, the sub-band at Fermi level is non-degenerate at *B*=0; at relatively high magnetic field, the degeneracy between Q and Q' valleys is further lifted by the valley Zeeman effect. It follows that an LL sextet can be lifted into two LL triplets caused by the valley Zeeman effect. For even-layer samples, the sub-band at Fermi level is spin-degenerate at *B*=0; at relatively high magnetic field, the degeneracy between up and down spins is lifted by the spin Zeeman effect. It follows that an LL duodectets can be lifted into LL sextets caused by the spin Zeeman effect.

Figure 4a,b show the calculated band structures of 3L MoS_2_ and 6L WS_2_ (see Supplementary Fig. 7 for the band structures of 3L WS_2_ and 6L MoS_2_), in which the minima of the conduction bands are not located at the K/K' points, but rather at the Q/Q' points, that is, between K(K') and Γ points, with quadratic sub-bands. As illustrated in Fig. 4c, 3 Q and 3 Q' valleys exist in the first Brillouin zone of the few-layer TMDCs. The C_3_ rotational symmetry dictates the threefold Q-valley degeneracy. For even-layer TMDCs, the Q and Q' valleys are further related by both time reversal and spatial inversion symmetries, which require Kramers degeneracy. Consider the low carrier density in our 6L WS_2_ device, the Fermi energy is ∼2.9 meV above the valley edge and crosses only one spin-degenerate sub-band at each valley (see the inset of Fig. 4b). Thus, in the SdH oscillations, we observe a twelve-fold LL degeneracy at low fields and sixfold LL degeneracy at high fields caused by the spin Zeeman splitting within each valley, that is, between |Q↑> and |Q↓> states (see Fig. 4d). The valley Zeeman effect is absent because of the inversion symmetry.

In contrast to the even-layer case, the inversion symmetry in the odd-layer devices is intrinsically broken; thus, all the sub-bands at each Q valley are spin non-degenerate. In our 3L MoS_2_ device (Fig. 4a), for instance, the Fermi energy is ∼6.0 meV above the valley edge and crosses only the lowest sub-band, for which the spin-up and spin-down sub-bands are lifted by 4.3 meV. Thus, the SdH oscillations exhibit sixfold LL degeneracy, which is reduced to threefold when the Zeeman effect is large, as shown in Fig. 4d. Although both the spin-up and spin-down sub-bands contribute to the quantum transport, the beating pattern cannot be observed since they originate from the same sub-band and have the same effective mass. The Zeeman effect in this case is obviously a valley Zeeman splitting between |Q↑> and |Q'↓> states, as a linear combination of the spin, orbital and lattice Zeeman effects. Despite the complex orbital hybridizations and the strong spin–orbital couplings, because of time reversal symmetry orbital and spin characters are opposite for the Q and Q' valleys, which can be split in the presence of *B* field. The lattice Zeeman effect arises from the opposite Berry curvatures of Bloch electrons at two valleys, which is also dictated by time reversal symmetry. In the inversion symmetric even-layer cases, the Berry curvature vanishes and so does the lattice Zeeman effect. Nevertheless, the Q/Q' valley Zeeman effect, observed here for the first time in transport, is analogous to the K/K' valley Zeeman effect observed using optical circular dichroism^13^,^14^,^15^,^16^.

## Discussion

Our realization of high-mobility TMDC devices and observation of their odd-even layer-dependence in the SdH oscillations, as well as the appearance of the onset of QH plateaus, are just the beginning for fully understanding the QH effects in atomically thin TMDCs. The existing theories focus exclusively on the K/K' valleys of monolayers. However, the quantum transports at low density for multilayers are dominated either by the electrons at Q/Q' valleys or the holes at the Γ valley. As there is only one quadratic sub-band across the Fermi level per Q valley in our current study, we are able to use the symmetry arguments and the well-known physics of the quadratic band to understand the observed even–odd layer-dependent behaviours in Zeeman effects. Further theoretical efforts are necessary in order to fully understand the quantum transports in atomically thin TMDCs. On the experimental side, the mobility of WS_2_ samples often appears higher than those of MoS_2_ samples; however, the observed SdH oscillations in MoS_2_ samples appear more pronounced. The observed SdH oscillations in odd-layer WS_2_ also appear weaker than that of even layers. Higher mobility and clearer knowledge of the stacking orders will be crucial for future systematic observations of QH effects in atomically thin TMDCs.

In summary, we demonstrate high-mobility TMDC FE transistors achieved by encapsulating atomically thin TMDCs between BN sheets. At moderate magnetic fields of 2.5–4 T and relatively low carrier density ∼10^12^ cm^−2^, the quantum oscillations are dominated by the Q valleys, exhibiting a universal even–odd layer dependence. Above 4 T, we observe spin Zeeman effects in even-layer devices and valley Zeeman effects in odd-layer devices. We also observe the onset of QH plateaus in the 3L device. The high-quality atomically thin BN-TMDC-BN-based FE transistors fabricated in this work pave the way for understanding the multi-valley band structures of few-layer TMDCs and for exploring their spin-valley entangled unconventional QH effects^33^.

## Methods

### Materials

MoS_2_ and WS_2_ crystals are grown by the chemical vapour transport method reported previously^34^. A mixture of Mo/W and S is first annealed in a sealed quartz tube at ∼800 °C. Then the polycrystalline powders together with iodine are annealed in a furnace (950 °C for the cold zone and 1,050 °C for the hot zone) for several weeks to grow large single crystals^35^,^36^. The as-grown 2H-type crystals are pre-examined by optical approaches before making FE transistors. The h-BN sources (Polartherm grade PT110) are bought from Momentive and annealed at a high temperature to improve the quality of BN crystals.

### Sandwiched heterostructures

To eliminate impurities induced during device fabrication, we employ a polymer-free dry transfer technique in an inert environment of argon or nitrogen, as schematically demonstrated in Supplementary Fig. 8a,b. Atomically thin flakes are mechanically exfoliated on 300 nm SiO_2_/Si substrates by the scotch-tape microcleavage method. A selected FL XS_2_ (*X*=Mo/W) is picked up from the SiO_2_/Si substrate by a thin h-BN flake (5–15 nm thick) on PMMA (950 A7, 500 nm) via van der Waals interactions. The h-BN/XS_2_ flake is then transferred onto a fresh thick h-BN flake at 40–50 °C, which is exfoliated previously on a different SiO_2_/Si substrate, to form a BN-XS_2_-BN heterostructure. The BN-XS_2_-BN structure and the high-temperature annealing can guarantee the stability of our XS_2_ devices. In the annealing process (conducted in Ar atmosphere above 300 °C), the small bubbles formed at the interfaces between h-BN and XS_2_ are largely removed and the charge trap density are largely reduced.

### Thickness determination

The thicknesses of XS_2_ flakes are characterized by atomic force microscopy (AFM, Veeco-Innva) as illustrated in Supplementary Fig. 9. The measured sample thicknesses *d*_m_ and their numbers of layers are listed in Supplementary Table 2 (ref. 37, 38). Technically, the roughness of SiO_2_ substrate surface is ∼1 nm, so there is a large variance when measuring the thickness of TMDCs lying on SiO_2_/Si substrates. In this study, we use a different method to determine the sample thickness (see Supplementary Fig. 9). We leave a small part of WS_2_ uncovered (without the top BN) when making the sandwiched structure. The bottom BN layer provides a smooth background in the AFM signal. The variance of sample thickness is smaller than 0.1 nm and the accuracy of the calculated number of layers is thus ensured.

### Selective etching process

To fabricate the metal electrodes, a hard mask is patterned on the BN-XS_2_-BN heterostructure by the standard electron-beam (e-beam) lithography technique using PMMA 950 A5 (see Supplementary Fig. 8c,d). Since the etching rate of XS_2_ by our reactive ion etching (CHF_3_:O_2_=4:40 s.c.c.m.) is lower than that of h-BN, the exposed top BN layer is then etched and XS_2_ is partially exposed. The electrodes are then patterned by e-beam lithography followed by O_2_ plasma etching (to remove PMMA residues) and standard e-beam evaporation (Ti/Au). Figure 1a–d show the schematic and the optical images of a typical BN-XS_2_-BN device with Hall-bar configurations. After the metal electrode deposition, the contact resistance is further reduced by a post annealing treatment at 300 °C in ambient pressure for ∼12 h.

### Transport measurement

The *I*_SD_–*V*_SD_ curves are measured by Keithley 6430. Other transport measurements are carried out using the standard lock-in technique (SR 830 with SR550 as the preamplifier and DS 360 as the function generator) in a cryogenic system. The cryogenic system provides stable temperatures ranging from 1.8 to 300 K and magnetic fields up to 9 T. For comparison, the MoS_2_ device with graphene electrodes are fabricated and the device shows a complicated feature (see Supplementary Fig. 10). However, our MoS_2_ FE transistors connected by standard metal electrodes produce clean data which can be explained by DFT calculations fairly well. Since we use the same quality of MoS_2_ channel material to fabricate these two kinds of devices, we conclude that MoS_2_-graphene-mixed devices always display complicated oscillations of magnetotransport which cannot be understood yet.

### Data availability

The data that support the findings of this study are available from the authors upon request.

## Additional information

**How to cite this article:** Wu, Z. *et al*. Even-odd layer-dependent magnetotransport of high mobility Q-valley electrons in transition metal disulfides. *Nat. Commun.* **7,** 12955 doi: 10.1038/ncomms12955 (2016).

## Supplementary Material

Supplementary Information Supplementary Figures 1-10 and Supplementary Tables 1-2
