# Supplementary material for: Even–odd layer-dependent magnetotransport of high-mobility Q-valley electrons in transition metal disulfides
Source: Nat Commun. 2016 Sep 21;7:12955. doi: 10.1038/ncomms12955 (PMC5036047; doi:10.1038/ncomms12955)
Supplement: Supplementary Information — Supplementary Figures 1-10 and Supplementary Tables 1-2 [file ncomms12955-s1.pdf]

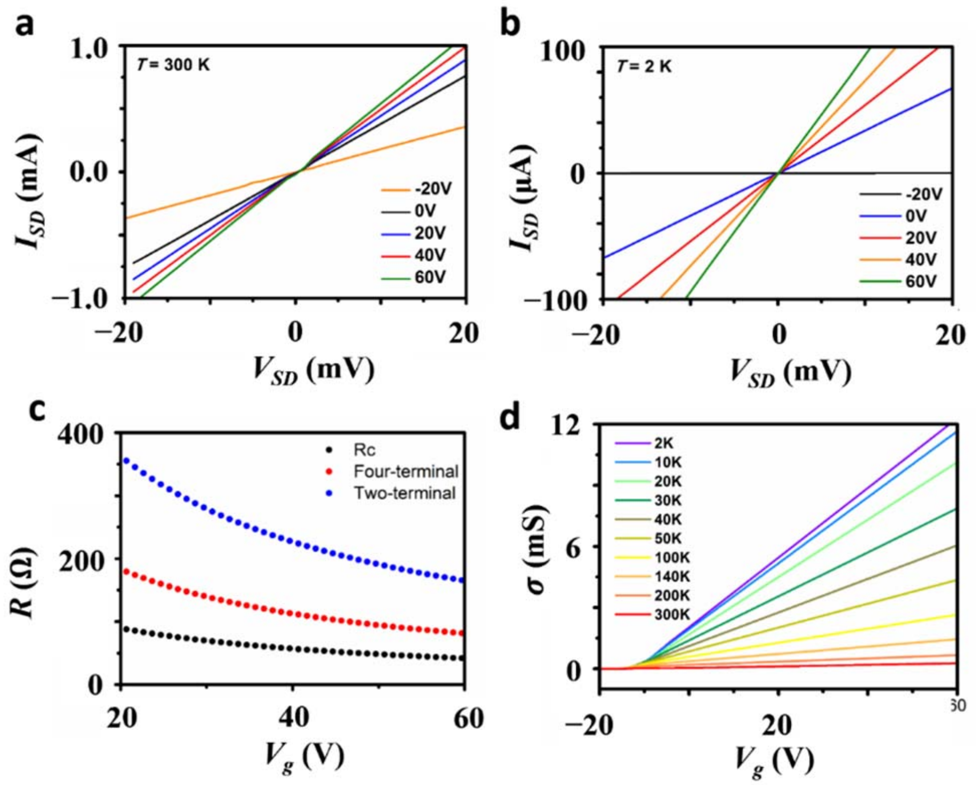

Supplementary Figure 1 | Output behavior and contact resistance of the 6L WS_2. (**a,b**) Two-terminal I_{SD}-V_{SD} characteristics for the WS_2 device at room temperature (**a**) and at a low temperature of 2 K (**b**). Linear behavior is observed in both cases. (**c**) Four-terminal, two-terminal, and calculated contact resistance R_c for WS_2 at T = 2 K. (**d**) Four-terminal conductance in the WS_2 device plotted as a function of the gate voltage at various temperatures.

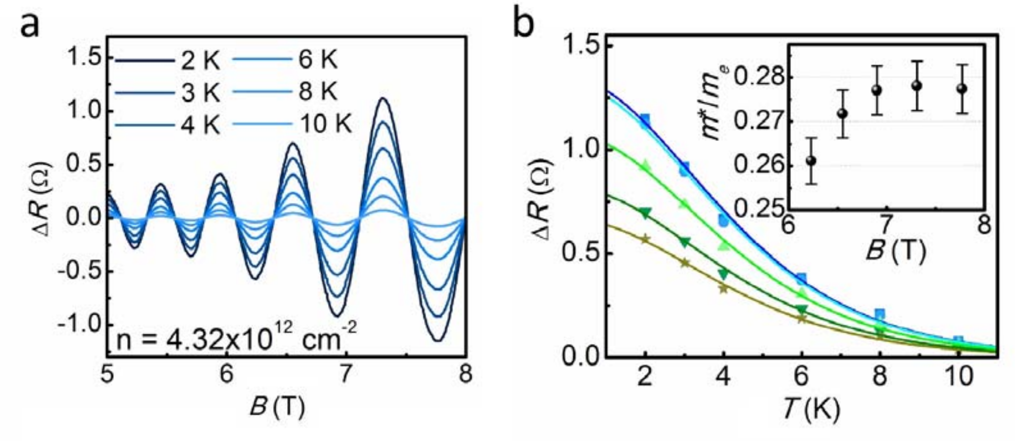

Supplementary Figure 2 | Temperature-dependent SdH oscillations of the 9L MoS_2. (**a**) ΔR plotted as a function of B at V_g = 60 V ($n = 4.32 \times 10^{12}$ cm^{-2}) at different temperatures. (**b**) Data points are the measured oscillation amplitude versus temperature T of the peaks at different B fields. The lines are fitted using the Lifshitz–Kosevich formula. The inset shows the fitted cyclotron mass (0.27 ± 0.01 m_e) at different B fields.

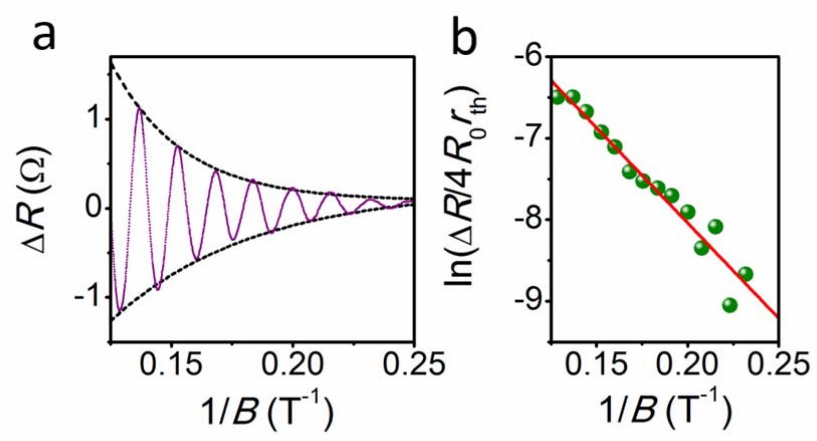

Supplementary Figure 3 | Quantum scattering time of the 9L-MoS_2. (**a**) The solid line is ΔR plotted as a function of inverse magnetic field B at V_g = 60 V and T = 2 K. The dash lines are the amplitude fitted using the Ando formula. (**b**) Dingle plot of ΔR for V_g = 60 V and T = 2 K, and the extracted quantum scattering time is 206 ± 6 fs.

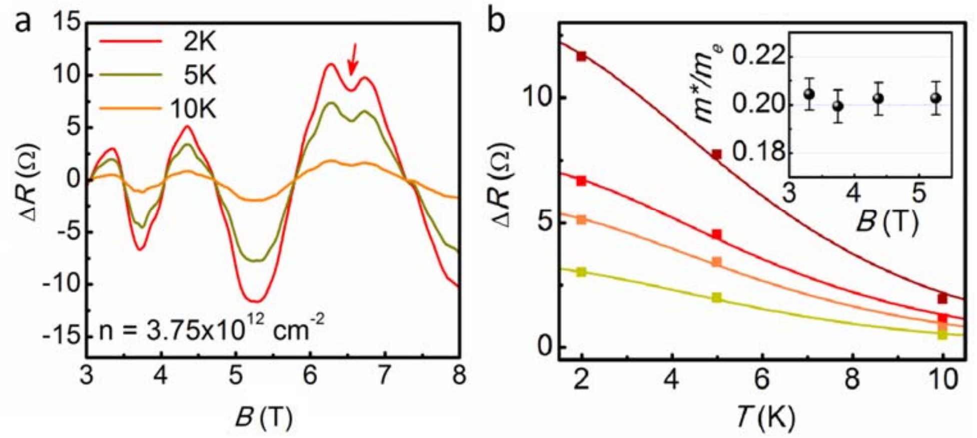

Supplementary Figure 4 | Temperature-dependent SdH ocillations of the 6L WS_2. (**a**) ΔR plotted as a function of B at V_g = 50 V ($n = 3.75 \times 10^{12}$ cm^{-2}) at different temperatures. The arrow shows the LLs with spin Zeeman splitting at high B fields. (**b**) Data points are the measured oscillation amplitude versus temperature T of the peaks at different B fields. The lines are fitted using the Lifshitz–Kosevich formula. The inset shows the fitted cyclotron mass (0.20 ± 0.01 m_e) at different B fields.

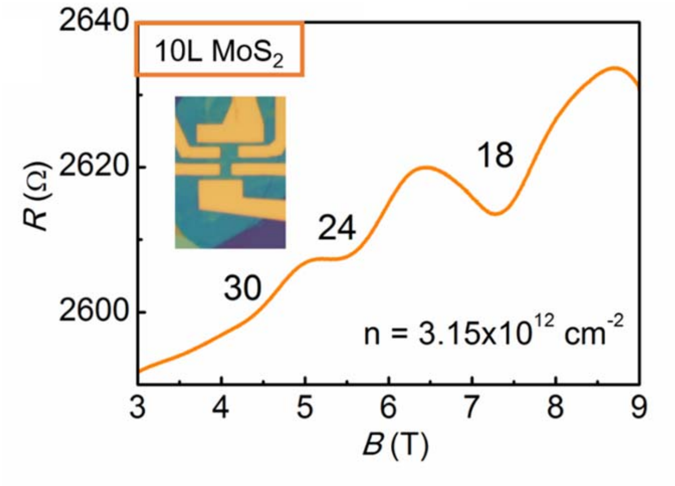

Supplementary Figure 5 | SdH ocilaations of the 10L MoS_2. The inset is the optical picture of the device.

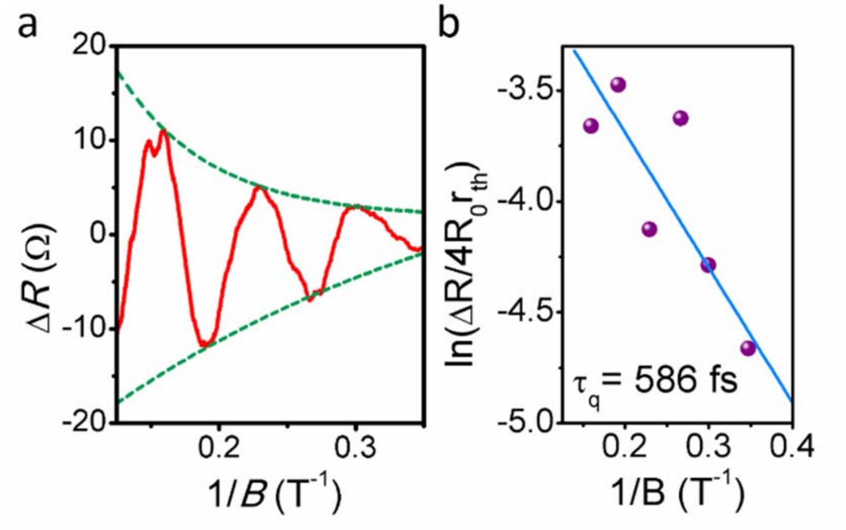

Supplementary Figure 6 | Quantum scattering time of the 6L-WS_2. (**a**) The solid line is ΔR plotted as a function of inverse magnetic field B at V_g = 50 V and T = 2 K. The dash lines are the amplitude fitted using the Ando formula. (**b**) The Dingle plot of ΔR for V_g = 50 V and T = 2 K yields a quantum scattering time of 586 ± 68 fs.

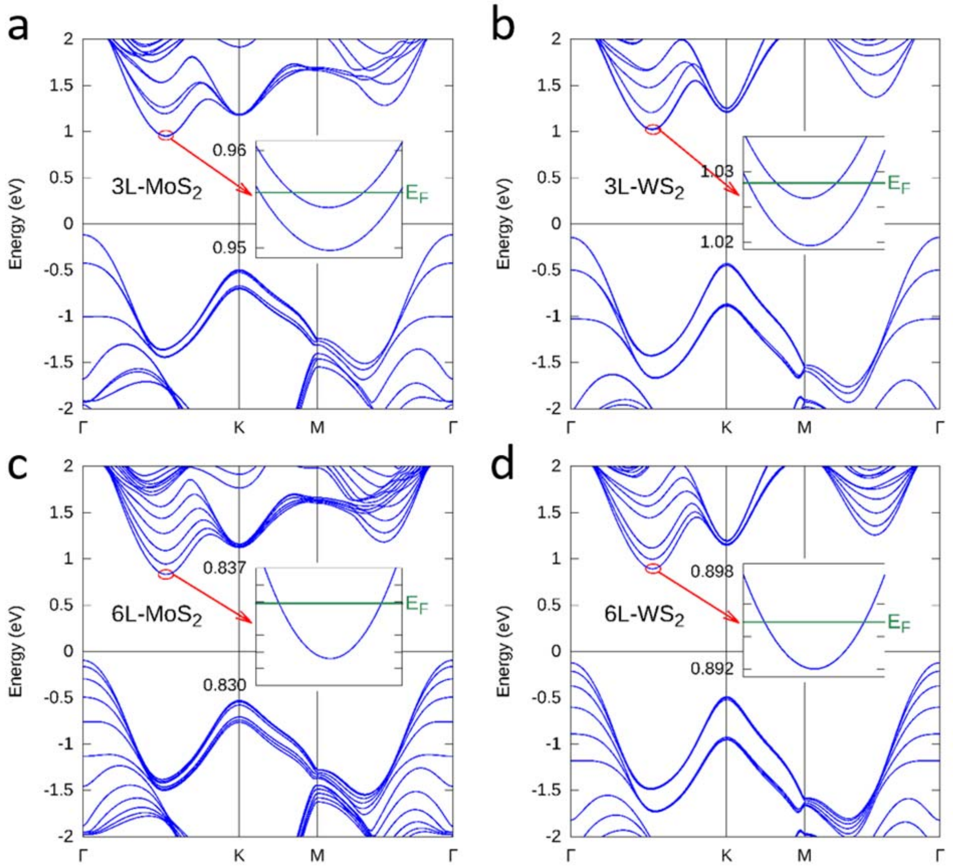

Supplementary Figure 7 | Calculated band structures of even- and odd-layer MoS_2 and WS_2.

(**a**) Calculated band structure of 3L MoS_2. The bottom of conduction band is located at the Q (Q’) valleys. At the edge of each Q (Q’) valley, the Fermi level only crosses the lowest non-degenerate sub-band, whose spin-up and spin-down sub-bands are lifted by 4.3 meV. (**b**) Calculated band structure of 3L WS_2. The Fermi level crosses the lowest non-degenerate sub-band, whose spin-up and spin-down sub-bands are lifted by 6.7 meV. (**c**, **d**) Calculated band structures of 6L MoS_2 and WS_2, respectively. The energy bands are spin degenerate at the edge of each Q (Q’) valley.

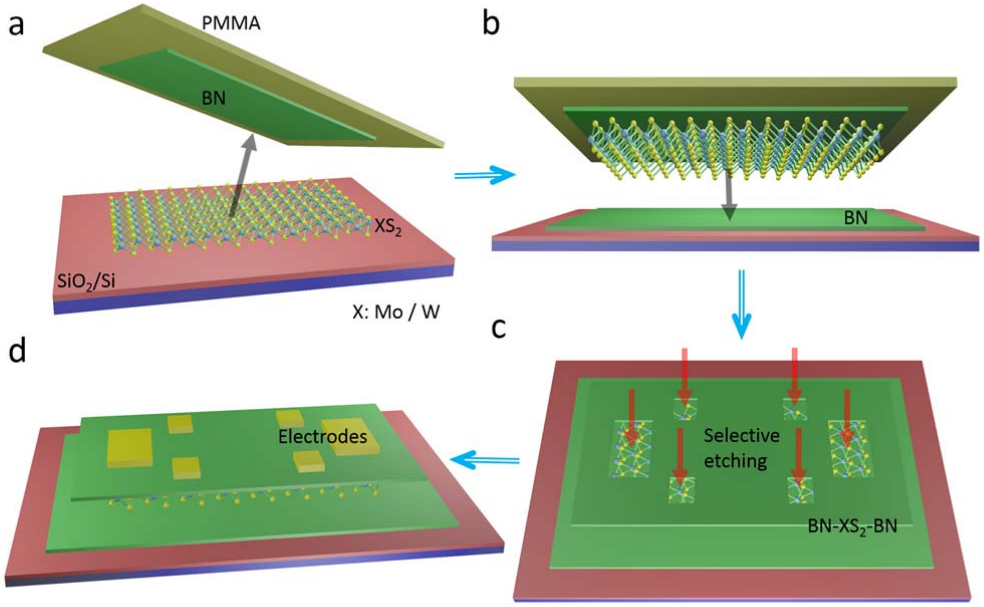

Supplementary Figure 8 | Fabrication of BN-XS_2-BN heterostructure device (X = Mo or W). (**a**) A selected few-layer XS_2 is picked up from the SiO_2/Si substrate by a thin h-BN flake (5-15 nm thick) via van der Waals interactions. (**b**) The h-BN/XS_2 flake is then transferred onto a fresh thick h-BN flake (to eliminate possible influence of the SiO_2 substrate), which is exfoliated previously on a different SiO_2/Si substrate, to form a BN-XS_2-BN heterostructure. (**c**) The exposed top BN layer is then etched by reactive ion etching and XS_2 is partially exposed. (**d**) The electrodes are then patterned by e-beam lithography followed by standard e-beam evaporation.

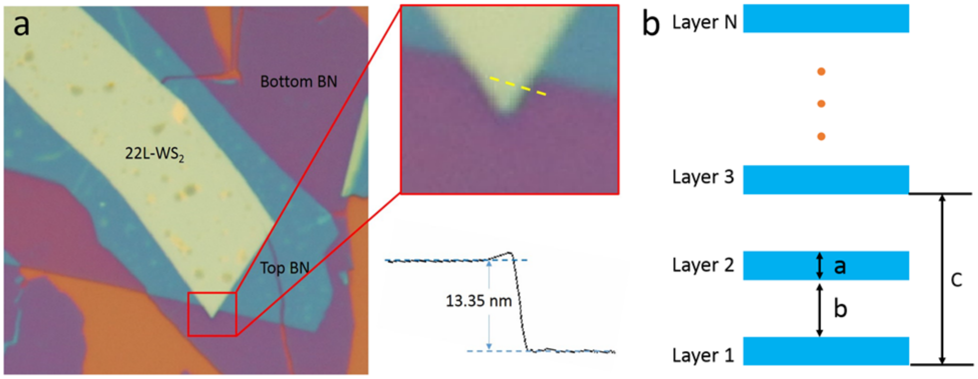

Supplementary Figure 9 | AFM characterization and schematic illustration of crystal structure. (a) Optical image of a BN-WS_2-BN sandwiched heterostrcuture on SiO_2/Si substrate. We leave a small part of WS_2 uncovered (without the top BN) when making the sandwiched structure. The bottom BN layer provides a smooth background in the AFM signal. (b) Schematic sectional view of FL TMDCs. Here a is the layer thickness, b is the layer spacing, $c = 2a + 2b$ is the lattice constant.

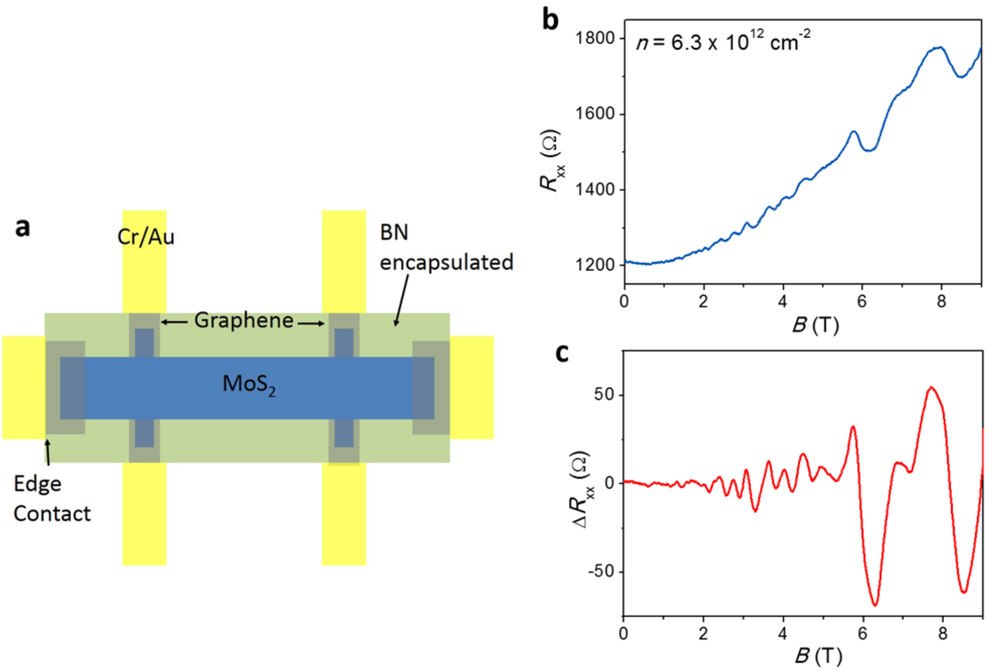

Supplementary Figure 10 | MoS_2 Hall bar device with graphene electrodes. (a) Schematic of the h-BN encapsulated MoS_2 multi-terminal device, with FL graphene as terminal electrodes. (b) Measured magneto-resistance R_{xx} at the carrier density of 6.3×10^{12} cm^{-2}. After subtracting the baseline, ΔR_{xx} shows a complicated feature with non-uniform oscillation periods.

Supplementary Table 1 | Lande factors of even- and odd-layer TMDs.

Sample No.	Lande factor
9L-MoS_2	3.4
6L-WS_2	2.2

The Lande factors were estimated by the temperature-dependence of SdH oscillations using the formula $g_L \mu_B B_C = kT_C$, where μ_B is the Bohr magneton, and B_C is the lowest field and T_C is the highest temperature for our observation of the Zeeman effect.

Supplementary Table 2 | Sample thicknesses and the number of layers.

	**a (nm)**	**b (nm)**	***d*=Na+(N-1)b (nm)**	**d_m (nm)**
**9L-MoS_2**	0.3172	0.2975	5.2348	5.37
**3L-MoS_2**			1.5466	1.62
**10L-MoS_2**			5.8495	5.90
**6L-WS_2**	0.3160	0.3015	3.4035	3.47
**10L-WS_2**			5.8735	5.90
**22L-WS_2**			13.2835	13.35
